# Aging Behavior and Mechanism Evolution of Nano-Al_2_O_3_/Styrene-Butadiene-Styrene-Modified Asphalt under Thermal-Oxidative Aging

**DOI:** 10.3390/ma16175866

**Published:** 2023-08-27

**Authors:** Zhiyuan Ji, Xing Wu, Yao Zhang, Gabriele Milani

**Affiliations:** 1 College of Architectural Science and Engineering, Yangzhou University, Yangzhou 225100, China; jzy@sinoroad.com; 2 Department of Architecture Built Environment and Construction Engineering, Politecnico di Milano, Piazza Leonardo da Vinci, 32, 20133 Milan, Italy; xing.wu@polimi.it (X.W.); gabriele.milani@polimi.it (G.M.)

**Keywords:** aging behavior, mechanism evolution, nanoalumina, SBS asphalt, thermal-oxidative aging

## Abstract

The goal of this paper is to analyze the aging behavior and the mechanism evolution of nano-Al_2_O_3_ (NA)-reinforced styrene-butadiene-styrene (SBS) asphalt under different thermal-oxidative aging conditions. First, NA/SBS-modified asphalt and SBS-modified asphalt with different aging levels were prepared. Second, the viscosity and high temperature rheological performance of the specimens were tested and the property-related aging indexes were calculated and compared. Third, a Fourier transform infrared (FTIR) test of the specimen was conducted and the chemical group-related aging indexes were calculated and analyzed. Fourth, gel permeation chromatography (GPC) was used to analyze the molecular weight of the specimens under different aging levels. Then, an atomic force microscope (AFM) was adopted to analyze the microsurface morphology of different specimens. Finally, correlation analysis between property-related indexes and chemical group indexes was conducted. The results show that NA can enhance the thermal-oxidative aging resistance of SBS asphalt. NA can inhibit the increase in sulfoxide groups and the degradation of the SBS polymer with the increase in aging. NA can slow down the formation of large molecule during the aging process. The degree of change in both the bee structures and micromorphological roughness of NA/SBS asphalt is lower than that of SBS asphalt under different aging levels.

## 1. Introduction

Asphalt concrete has excellent road performance and is widely used for highway pavement construction due to its convenient construction and maintenance [1,2]. Asphalt is a key material in asphalt concrete, which directly affects the performance of asphalt pavement [3]. However, during the utilization period of asphalt pavement, complex environmental conditions and traffic loads can lead to various road distresses. Researchers have indicated that thermal-oxidative aging causes irreversible damage to asphalt properties, and is one of the main reasons for road distresses [4]. Specifically, the thermal-oxidative effect could first decrease the performance of the asphalt, which decreases the adhesion ability and crack resistance of asphalt material [5]. Then, it induces other road distresses, such as cracking, etc.

SBS asphalt is widely used in the construction of high-grade road surfaces due to its excellent performance. However, SBS asphalt also undergoes noticeable aging under thermal-oxidative conditions [6]. Compared to the aging of base asphalt, the aging of SBS asphalt is more complex. It involves both the hardening phenomenon of base asphalt and the degradation of the SBS polymer [7]. The significant performance deterioration of SBS asphalt under thermal-oxidative aging conditions has been a concern among researchers.

There are studies indicating that the chemical groups in the polymer chains of SBS asphalt are highly sensitive to thermal oxidation conditions [4,8]. Hao et al. investigated the influence of aging conditions on the aging characteristics of SBS asphalt [9]. Cortizo et al. [10] believed that the formation of free radicals from chain scission could lead to an increase in polar compounds in SBS asphalt. Wei et al. [11] studied SBS asphalt from the perspective of microstructure and molecular weight, and found reactions such as oxidation, chain breakage, and large polymer cluster degradation in the aging process. Ruan et al. [12] believed that thermal-oxidative aging disrupted the polymer network structure, and as aging time increased, the polymer gradually degraded. Liu et al. [13] used the dynamic shear rheometer (DSR) test to test the rheological properties of aged SBS asphalt and characterized the changes in molecular weight before and after aging. 

Considering the irreversible damage caused by asphalt aging on the performance of asphalt pavement, it is important to enhance its long-term resistance to thermal-oxidative aging. Numerous studies have utilized different modifiers to enhance the aging resistance of SBS asphalt, such as fibers, anti-rutting agents, etc. The application of nanomaterials in road engineering has been widely reported [14]. Sun and his colleagues believed that nanomaterials had the potential to be used as asphalt modifiers and conducted extensive experimental research on nanomaterial-modified asphalt, including the construction of test roads [15,16]. Ren et al. summarized and analyzed research into using nanomaterials in asphalt and attempted to modify bio-oil asphalt using five different nanomaterials. The experimental results showed improvements in the aging resistance of bio-oil asphalt [17]. Yadykova A. Y. et al. studied the influence of bio-oil and silica nanoparticles on the adhesion performance of asphalt. According to the adhesion performance test results, 5% bio-oil was recommended as the optimal mixing amount. It was found that hydrophobic silica could better improve asphalt adhesion performance than hydrophilic silica [18]. Yadykova A. Y. et al. also conducted a detailed study on bio-oil and nanomaterials, and their analysis revealed that the addition of 10% bio-oil could significantly improve the adhesion of asphalt to the maximum extent. Additionally, hydrophobic nanoclay enhanced the elasticity and adhesion of asphalt by inducing gelation [19]. Some researchers used metal oxide nanoparticles (nano-SiO_2_, nano-TiO_2_, nano-ZnO) to study the reinforcing effect of them on the aging resistance of SBS asphalt [20]. Zhang et al. [21] utilized rheological tests and aging tests and discovered that nano-SiO_2_ had a positive effect on improving the rheological properties and aging resistance of asphalt. Nano-ZnO was reported multiple times to enhance the aging resistance of asphalt [22,23]. Li et al. [24] found that nano-ZnO could reduce the mass change and viscosity aging index during asphalt aging, which means it improved the aging resistance of asphalt. Wang et al. [25] reported excellent aging resistance properties of carbon nanotubes/SBS composite-modified asphalt. 

Among all these nanomaterials, nano-Al_2_O_3_ (NA) has also been experimented with to modify asphalt due to its excellent high-temperature and chemical stability. Al-Mansob et al. [26] used NA as a modifier to enhance the properties of epoxy-modified natural rubber asphalt (ENRMA). Similarly, Ali and Shafabakhsh et al. [27,28] discovered that NA improved the high temperature properties and storage stability of asphalt. Bhata et al. [29] found that NA had the potential to enhance the aging resistance of SBS-modified asphalt. On the other hand, the potential of NA to modify polymer materials is attractive because NA plays a positive role in the modification and reinforcement of polymers, especially in the enhancement of polymer stability [30,31]. However, the current research on the antioxidant aging resistance of SBS asphalt modified by NA is not comprehensive, and the important mechanism of thermal-oxidative aging resistance of NA-reinforced SBS asphalt has been ignored.

Hence, it is of great significance to conduct a specialized study on the thermal-oxidative aging characteristics of NA/SBS modified asphalt, and to investigate the important aging strengthening mechanisms of NA in SBS asphalt. An increasing number of studies have adopted the approach of extending the aging test duration to examine the characteristics of asphalt thermal-oxidative aging [32]. Nagabhushanarao et al. [33] simulated long-term thermal-oxidative aging conditions by performing multiple cycles of the rolling thin film oven test (RTFOT). Ye et al. [34] conducted a comprehensive analysis of the effects of different durations and temperatures of the RTFOT on asphalt performance and concluded that aging had the most significant impact on asphalt fatigue performance. Based on a previous analysis, Yu et al. found that the prolonged aging time of the standard RTFOT could simulate long-term aging. Therefore, the standard aging time was extended to study the stability and aging characteristics of 15 asphalt samples under different aging times. Then, the destruction of the polymer network could be observed during the aging process [35]. Ibrahim, B. et al. extended the duration of the RTFOT test to 8 days to simulate the long-term aging of asphalt and studied the effect of antioxidants on asphalt performance. They recommended 10% crepe rubber and 2% trimethyl-quinoline as the optimal admixture combination based on the results in asphalt performance improvement [36]. Siddiqui et al. investigated the performance of four types of asphalt after undergoing multiple cycles of RTFOT aging. The results indicated that the asphalt aged through four cycles of the RTFOT exhibited similar rheological properties to those aged for an extended period of time [37]. At present, the approach of extending the aging time to study the aging characteristics of asphalt has become a common and effective research method.

Hence, in this study, we chose to perform multiple cycles of the RTFOT test (the first, second, third, fourth, and fifth cycles) to simulate the thermal-oxidative aging process from short-term aging to long-term aging.

Therefore, this paper first focuses on a detailed evaluation of the influence of NA on the aging property of SBS asphalt under different thermal-oxidative aging levels. Then, a comprehensive analysis of the thermal-oxidative aging mechanism of NA/SBS composite-modified asphalt is analyzed from the perspectives of its chemical functional groups, molecular weight, and micromorphology. Specifically, the NA/SBS-modified asphalt and SBS asphalt under different aging levels were prepared by changing the RTFOT cycles. Second, the rheological properties of the samples were tested and analyzed. Third, Fourier transform infrared spectroscopy (FTIR), gel permeation chromatography (GPC), and atomic force microscopy (AFM) were used to observe the chemical functional groups, molecular weight distribution, and micromorphology evolution of the tested asphalt under different thermal-oxidative aging levels. This paper could help to evaluate the reinforcing effect of NA on the long-term thermal-oxidative aging property of SBS asphalt and to reveal the anti-aging strengthening mechanism of NA in SBS asphalt.

The innovations of this study are as follows:

1. To evaluate the influence of NA on the thermal-oxidative aging properties of SBS asphalt, and to carry out research on the aging characteristics of NA/SBS-modified asphalt under long-term thermal-oxidative aging conditions.
2. To reveal the enhancement mechanism of NA/SBS under thermal-oxidative aging conditions from the perspective of chemical functional groups, molecular weight, and micromorphology.

## 2. Materials and Methods

### 2.1. Materials

The raw materials used to prepare the SBS asphalt and the NA/SBS-modified asphalt were base asphalt, linear SBS polymer, and nano-Al_2_O_3_ (NA). The base asphalt and SBS polymer were produced by China Petroleum & Chemical Corporation (Beijing, China), while the NA was manufactured by Zhejiang Hengna Co., Ltd. (Hangzhou, China). The physical properties of these materials are presented in Table 1.

### 2.2. Sample Preparation

The samples prepared in this paper can be divided into two categories, namely SBS asphalt (SBSAB) and NA/SBS composite-modified asphalt (NASBS). First, 5% SBS polymer (by weight of base asphalt) was added into the base asphalt to prepare the SBS asphalt. The linear SBS modifier was added into the hot base asphalt at 175 ± 5 °C. A high-speed shear mixer was used to mix them together at a rate of 6000 r/min for 1 h. Then, the SBS asphalt samples were aged using the rolling thin film oven test (RTFOT) under different standard aging cycles (cycles 0, 1, 2, 3, 4, and 5). 

In the research on using NA to modify other types of asphalt, the suggested NA content is 5% (by weight of SBS asphalt), because the NA particles might agglomerate with each other when their content is higher than 5% [38,39]. Therefore, this paper adopted 5% of the weight of SBS asphalt as the content of NA to prepare the NA/SBS-modified asphalt. NA was added into the SBS asphalt at 175 ± 5 °C, and they were mixed using a high-speed shear mixer at a rate of 6000 r/min for 1 h. After preparing the NASBS, the samples were also aged using the RTFOT under different standard aging cycles (cycles 0, 1, 2, 3, 4, and 5). The samples prepared in this paper are marked using codes to make it easier to understand. Samples’ information is shown in Table 2.

### 2.3. Experimental Methods

#### 2.3.1. Aging Procedure

The aging procedure adopted in this study is the rolling thin film oven test (RTFOT) aging method. It is a kind of thermal-oxidative aging procedure. During the aging procedure, aging cylinder bottles containing the test samples were placed in an oven, and the asphalt samples were ensured to maintain a thin film form. The temperature was set at 163 °C, and the flow rate of hot air was controlled within the range of 4000 mL/min ± 200 mL/min. The aging bottle was rotated at a speed of 15 r/min. The experimental procedures were conducted following ASTM D2872 [40]. 

The standard aging time of RTFOT aging is 85 min. Therefore, in order to study the aging behavior of the asphalt samples under different aging levels, the SBSAB and the NASBS were aged under different RTFOT cycles. The number of RTFOT cycles was selected as 1, 2, 3, 4, and 5 loading cycles for both the SBSAB and the NASBS. The aging times for the different aging cycles were 85 min, 170 min, 255 min, 340 min, and 425 min, respectively.

In order to quantify the impact of thermal-oxidative aging on the properties of the asphalt samples, different aging indexes were used to characterize the aging behavior of the samples. The specific aging indexes are listed in the following sections.

#### 2.3.2. Viscosity Test

The viscosity (η) of the asphalt samples under different aging levels was measured using a Brookfield rotational viscometer. This index evaluated the flowability of the asphalt samples. Experimental procedures were conducted following ASTM D4402 [41]. In each test, the asphalt samples were tested at 135 °C. The measurement device used for the viscosity testing were coaxial cylinders, with a specification of 27#. The shear rate was set to 50.00 s^−1^. The aging index of *η* used in this study was the viscosity aging index (*VAI*), and it was calculated using Equation (1).

(1) $$VAI=\frac{\eta_{Aged}}{\eta_{Unaged}}$$

#### 2.3.3. DSR Test

A DSR test was used to evaluate the rheological properties of the asphalt samples. The asphalt samples were subjected to periodic shear loading during the test, which was able to provide the parameters of the asphalt’s rheological properties to assess its deformability, etc. The rheological property indexes used in this paper were complex modulus (G*) and rutting factor (G*/*sinδ*). The specific experimental procedures were listed in the specifications outlined in AASHTO T315 [42]. The angular frequency and strain parameters were set to 10 rad/s and 12%, respectively.

The aging indexes of G* and G*/*sinδ* are called the complex modulus aging index (*CAI*) and rutting factor aging index (*RFAI*), respectively. The aging indexes were calculated using the test data at 64 °C, and the indexes were calculated using Equations (2) and (3).

(2) $$CAI=\frac{{G^{*}}_{Aged}}{{G^{*}}_{Unaged}}$$

(3) $$RFAI=\frac{{G^{*}/sin\delta }_{Aged}}{{G^{*}/sin\delta }_{Unaged}}$$

#### 2.3.4. FTIR Test

The aging of asphalt can also be reflected in a change in internal chemical bonds or functional groups [43,44]. The Fourier transform infrared (FTIR) test has been widely used in asphalt research to analyze the chemical components of asphalt materials and can help researchers understand the chemical functional group information and the molecular structure of asphalt. Therefore, a FTIR test was adopted in this study to obtain the spectrum of the unaged and aged asphalt samples. The FTIR spectrums were then analyzed using EZOMNIC version 7.3 software to extract the representative characteristic peak information and indexes. The spectrums were baseline-corrected using EZOMNIC version 7.3, and then the peak area tool in the software was used to capture the peaks at the specified positions. The software automatically captured the characteristic peaks and calculated their area. The detailed introduction of the aging indexes related to the FTIR test is listed in the discussion section.

#### 2.3.5. GPC Test

Gel permeation chromatography (GPC) is an efficient liquid chromatography technique that could detect the molecular weight and accurately determine the molecular weight distribution of materials [45,46]. The principle of GPC analysis is to separate compounds using a gel column, where compounds with a higher molecular weight have faster penetration rates while compounds with a lower molecular weight have slower penetration rates. Compounds with a higher molecular weight are eluted first, and their signal appears first in the GPC test curve. Compounds with a lower molecular weight are eluted later, and their signal appears later. Asphalt and modified asphalt are a kind of complex blends composed of different molecules with different molecular weights. Each polymer with a different molecular weight inside the asphalt material has different contents. Therefore, GPC was used in this study to analyze the change in the molecular weight distribution of the asphalt materials before and after the aging procedure. Calibration is required for GPC testing, which involves testing standard samples with different molecular weights, constructing a standard curve based on the test results of the standard samples, and determining the equation of the standard curve.

**Figure 1:**
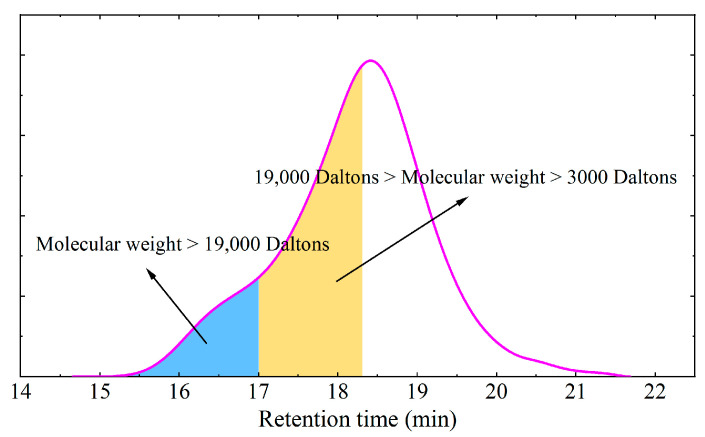
GPC test curve.

In this study, the experiments were conducted at room temperature. As is shown in Figure 1, after obtaining the curve of the retention time and the signal strength, the curve is divided into three components when analyzing the GPC test result. The first part is the molecule of which the weight is higher than 19,000 Daltons, and it is identified as the SBS polymer. The second part is the molecule of which the weight is between 3000 Daltons and 19,000 Daltons, and it is identified as asphaltene [9,47].

#### 2.3.6. AFM Test

Atomic force microscope (AFM) is an analysis technique that allows for the observation of the surface microstructures of materials, and it is commonly used to study surface morphology and roughness. The principle of AFM analysis involves obtaining surface topography by measuring the interaction forces between a non-contact probe and the sample surface, thereby providing detailed information about the surface microstructure features [48]. The probe type is Tap300, with a needle tip curvature radius of less than 10 nm. The test is conducted in tapping mode.

AFM offers advantages such as high resolution, high sensitivity, and the ability to operate under ambient conditions without the need for a vacuum environment. AFM experiments are widely applied in fields such as materials science, biomedical research, and nanotechnology to investigate surface morphology, nanoscale structures, and the three-dimensional conformation of biomolecules.

In this study, AFM was conducted at room temperature, and the Nanoscope Analysis software version 1.5 was used for further analysis of the AFM images to obtain the surface micromorphology and the microsurface roughness data of the asphalt samples.

### 2.4. Experimental Design

**Figure 2:**
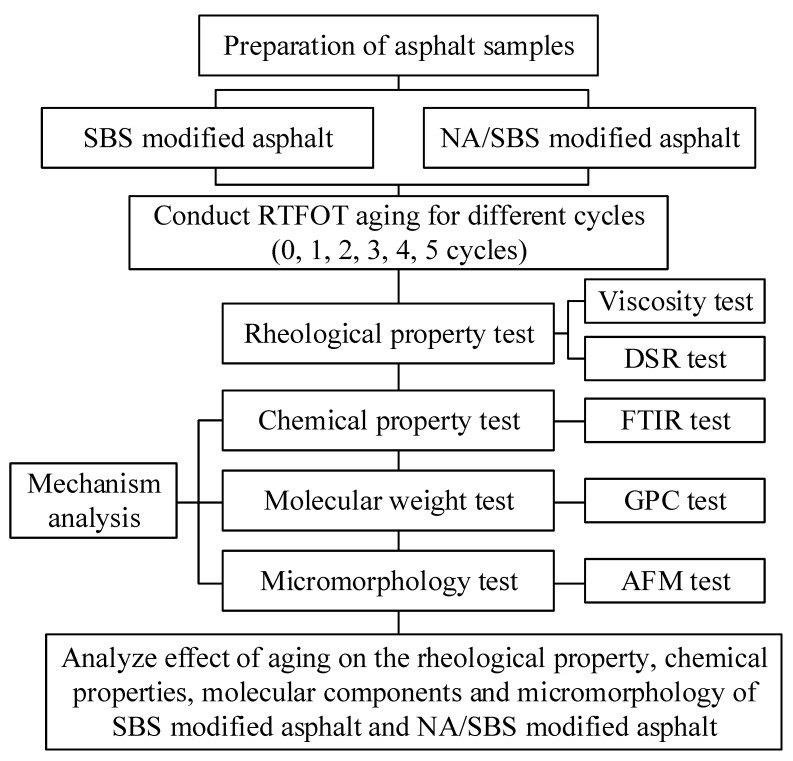
Flowchart of the experimental design.

The experimental design of this research is illustrated in Figure 2.

## 3. Results and Discussion

### 3.1. Rheological Property Test Results

#### 3.1.1. Viscosity Test Results

**Figure 3:**
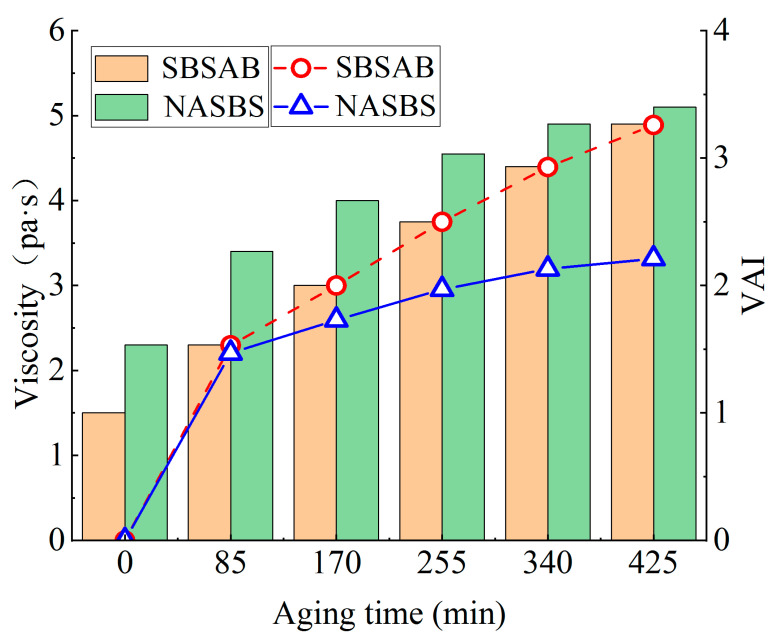
Viscosity (shear rate was set to 50.00 s^−1^) and viscosity aging index.

Figure 3 shows the viscosity and *VAI* values of the asphalt samples under different thermal-oxidative aging levels. As the aging time increases, both the SBSAB and NASBS asphalt samples exhibit an increasing trend in viscosity. The viscosity of SBSAB shows a maximum increase of 227%, while the viscosity of NASBS increases by 122%. Clearly, under the same duration of thermal-oxidative aging, NASBS exhibits a lower increase in viscosity compared to SBSAB, which is a positive outcome. 

The *VAI* quantifies the extent of viscosity changes caused by thermal-oxidative aging. Prolonged aging leads to an increase in the *VAI* in both asphalt samples. However, the *VAI* growth rate of SBSAB is significantly higher than that of NASBS. Throughout the entire long-term aging process, the *VAI* values of NASBS are consistently lower than those of SBSAB in each aging cycle. A lower *VAI* value indicates a lower impact of aging on asphalt flowability and better anti-aging performance. This suggests that the addition of NA significantly enhances the thermal-oxidative aging resistance of SBSAB, and this positive effect becomes more pronounced when the thermal-oxidative aging level is higher.

#### 3.1.2. DSR Test Results

**Figure 4:**
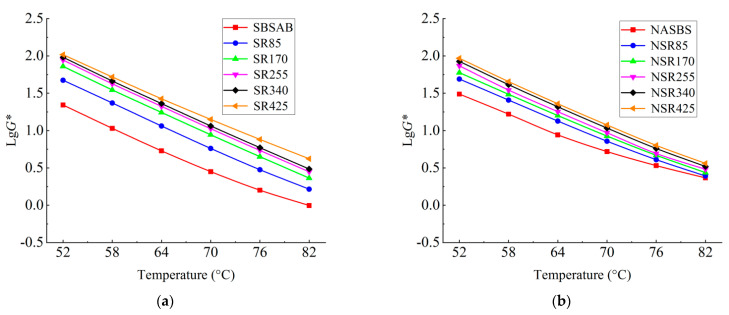
Complex modulus (angular frequency was set to 10 rad/s) of (**a**) SBS asphalt; (**b**) NA/SBS modified asphalt.

Figure 4a,b show the complex modulus G* of SBSAB and NASBS under different aging levels. The G* values for both SBSAB and NASBS increase with the increase in aging time. The G* value reflects the ability of asphalt materials to resist deformation, and the increase in G* is mainly attributed to the conversion of small molecular substances into larger molecular substances under the influence of high temperature and oxygen, which leads to an increase in asphalt stiffness. Under the same thermal-oxidative aging time and test temperature (64 °C), the increasing extent of the G* value of SBSAB is larger than that of NASBS. For example, the increases in the G* value of SBSAB during the first and fifth aging processes are 115% and 399%, respectively. The corresponding increasing extents for NASBS are 53% and 161%. This indicates that the addition of NA could delay the hardening effect of SBS asphalt under aging conditions. After comparing with other references, it could also be noted that the anti-hardening effect of NA is more significant than that of mesoporous silica nanoparticles [49]. 

**Figure 5:**
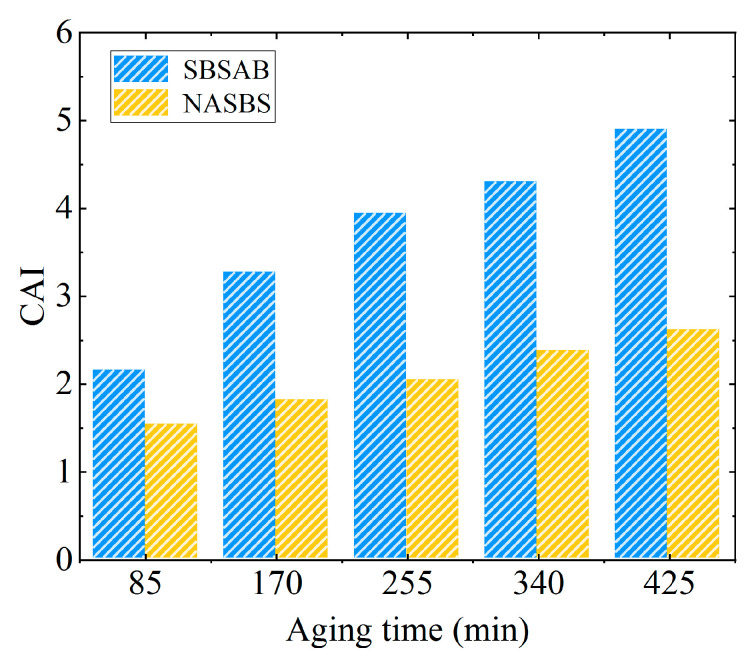
Complex modulus aging index.

It can be observed from Figure 5 that the *CAI* values for SBSAB and NASBS both increase with the increase of aging time. The increasing extent of the *CAI* of SBSAB is more significant than that of NASBS. When the aging cycle changes from 1 to 5, the *CAI* value for SBSAB increases from 2.15 to 4.9. The increasing extent is 128%. In contrast, the *CAI* value for NASBS increases from 1.53 to 2.61, and the increasing extent is 71%. It can be seen that the increasing extent of *CAI* of NASBS is lower than that of SBSAB. Meanwhile, the *CAI* value for NASBS remains lower than that for SBSAB throughout the entire range of the aging process. This also indicates that NA has a positive effect on the thermal-oxidative aging resistance of SBS asphalt.

**Figure 6:**
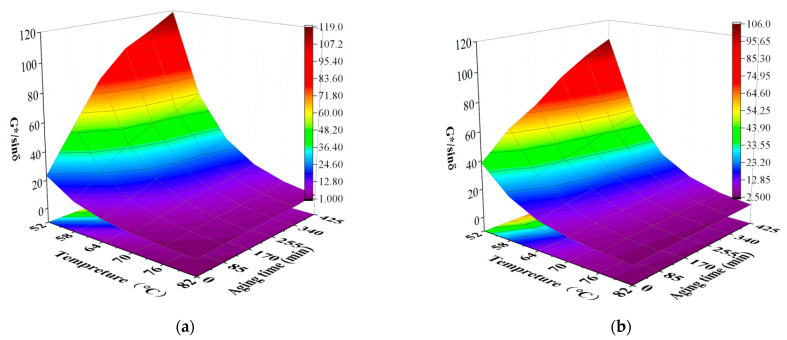
Rutting factor for (**a**) SBS asphalt; (**b**) NA/SBS modified asphalt.

Figure 6 illustrates the influence of thermal-oxidative aging time on the G*/*sinδ* of SBSAB and NASBS. Under the same thermal-oxidative aging time, the G*/*sinδ* values for both types of asphalt decrease as the test temperature increases. The G*/*sinδ* shows an increasing trend as the thermal aging time increases under each temperature. The increasing extent of the G*/*sinδ* of SBSAB is higher than that of NASBS. Thus, NASBS is more stable as the aging time increases.

**Figure 7:**
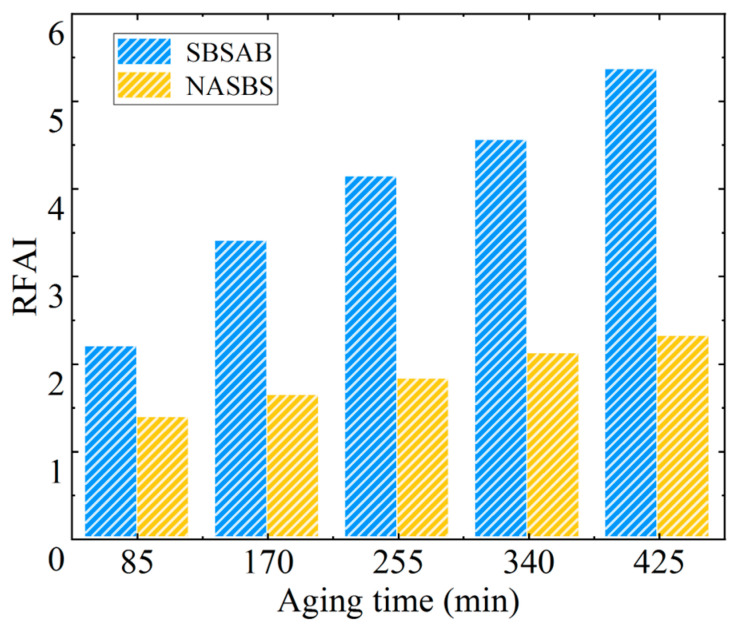
Rutting factor aging index.

As to the *RFAI* values shown in Figure 7, the *RFAI* values of both types of asphalt show a similar increasing trend as the aging time increases. It should be noted that the increasing extents of the *RFAI* of SBSAB and NASBS are 145% and 67%, respectively, when the aging time increases from 85 min to 425 min. Hence, the *RFAI* increasing extent of NASBS is much lower than that of SBSAB. Meanwhile, throughout the whole thermal-oxidative aging process, the *RFAI* value of NASBS remains lower than that of SBSAB, and the difference between them keeps increasing as the aging degree increases. In comparison with other research under the same level of aging, NA exhibits a more significant anti-aging effect compared to nano-zinc oxide and nanosilica particles [50,51].

Generally, the aging indexes of the rheological property tests showed that the anti-aging ability of NASBS was better than that of SBSAB, which indicated that NA could increase the anti-aging ability of SBS asphalt.

### 3.2. Mechanism Analyzing Test Results

Based on the analysis of the rheological property experimental data, it can be concluded that SBS asphalt modified with NA exhibits superior resistance to aging under thermal-oxidative conditions, and this positive effect becomes more pronounced with the increase in aging time. In the following sections, this paper focuses on investigating the underlying mechanisms of this phenomenon. It should be noted that three representative aging levels were adopted to analyze these mechanisms in SBSAB and NASBS, namely RTFOT aging cycles 0, 1, and 5.

#### 3.2.1. FTIR Test Results

**Figure 8:**
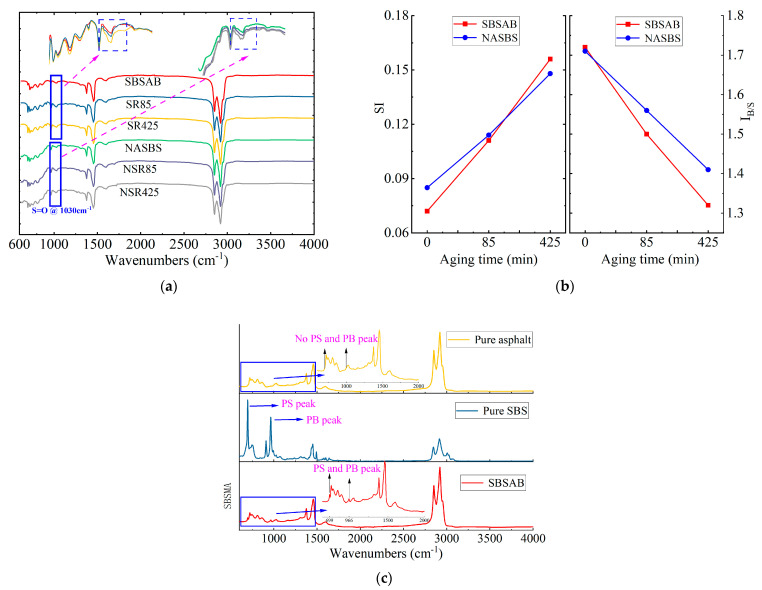
FTIR test results for (**a**) FTIR spectrums; (**b**) Sulfoxyl index and polymer index; and (**c**) SBS asphalt.

The Fourier transform infrared (FTIR) spectrums of SBSAB and NASBS under different aging cycles are shown in Figure 8a. The peak positions of the characteristic peaks in the spectrums of SBSAB and NASBS are basically the same, and there are no new absorption peaks formed. Specifically, the peaks at 1460 cm^−1^ and 1376 cm^−1^ are the in-plane bending vibration absorption peaks of aliphatic C-H groups. The characteristic absorption peak at 1030 cm^−1^ is the stretching vibration of the sulfoxide S=O group. The absorption peak at 966 cm^−1^ is the characteristic peak of the polybutadiene (PB) segment, and the absorption peak at 699 cm^−1^ is the characteristic peak of the polystyrene (PS) segment. Some chemical functional groups are affected by thermal-oxidative aging conditions. For instance, in the aging process, the peak absorbance at 1030 cm^−1^ increases dramatically, indicating that the amount of S=O functional groups in bitumen rises gradually. This change can be observed in the magnified portion of Figure 8 [52]. The SBS polymer contains unsaturated C=C bonds in its PB segment, which will degrade in a thermal-oxidative aging environment. Aging affects both carbonyl and sulfone groups, but upon comparison, sulfone groups are more significantly influenced by aging and are easier to quantify for analysis [53].

In analyzing SBS asphalt, some researchers adopt several aging indexes to quantitatively represent the aging degree of the asphalt. Sulfoxyl index (*SI*) and polymer index (*I_B_*_/*S*_) are selected as indexes to quantify the content of sulfoxide groups and the cracking effect of the SBS modifier in the asphalt material [54,55]. The calculation equations are listed in Equations (4) and (5).

(4) $$SI=\frac{A_{S=O}}{A_{{1456cm}^{-1}}}$$

(5) $$I_{B/S}=\frac{A_{PB}}{A_{PS}}=\frac{A_{{966cm}^{-1}}}{A_{{699cm}^{-1}}}$$

where $A_{S=O}$ represents the peak area centered at 1030 cm^−1^; $A_{{1456cm}^{-1}}$ represents the area centered at 1456 cm^−1^; $A_{PB}$ represents the peak area at 966 cm^−1^; and $A_{PS}$ indicates the peak area at 699 cm^−1^.

Figure 8b shows the *SI* values and *I_B_*_/*S*_ values of SBSAB and NASBS at different thermal-oxidative aging degrees. The *SI* growth rate of SBSAB is significantly higher than that of NASBS under long-term thermal oxidation aging levels. After undergoing 85 min and 425 min of thermal-oxidative aging, the *SI* values of SBSAB increased by 54% and 117%, respectively. On the other hand, the *SI* values of NASBS experienced an increase of 34% and 74% under the corresponding thermal-oxidative aging time. This phenomenon indicates that the addition of NA inhibits the growth of sulfoxide groups in NASBS during thermal-oxidative aging. 

The *I_B_*_/*S*_ values of SBSAB and NASBS decreased significantly after thermal oxidation aging, which was caused by the degradation of the SBS. The rate of *I_B_*_/*S*_ reduction in SBSAB was higher than that in NASBS. After undergoing 85 min and 425 min of thermal-oxidative aging, the *I_B_*_/*S*_ values of SBSAB decreased by 13% and 23%, respectively. The *I_B_*_/*S*_ values of NASBS reduced by 9% and 18% under the same thermal oxidation aging conditions, indicating that the SBS degradation in SBSAB was more severe during thermal oxidation aging. It can then be inferred that the NA in NASBS asphalt can alleviate the degradation of SBS under thermal oxidation aging conditions.

#### 3.2.2. GPC Test Results

**Figure 9:**
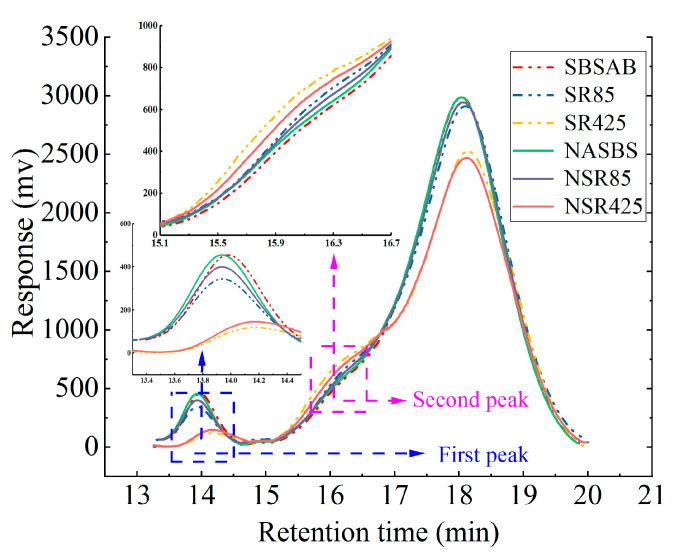
GPC test result.

Figure 9 shows the gel permeation chromatography (GPC) test results of SBSAB and NASBS. As is mentioned before, the SBS polymer is the first to be detected in a GPC test, followed by asphaltene and other smaller components inside the asphalt material. There are mainly two peaks in Figure 9. Therefore, the first peak is the characteristic peak of the SBS polymer and the second peak represents the asphaltene in asphalt material.

It can be seen in Figure 9 that the first peak of SBSAB and NASBS decreases after the 85 min aging procedure. This means that the SBS polymer degrades during this period, decreasing its content. After being aged for 425 min, the decreasing extent of the first peak of SBSAB and NASBS is more obvious, which shows that the increase in aging time makes the degradation of the SBS polymers inside the asphalt more severe. However, it can be noticed that the decreasing degree of the first peak of NASBS is lower than that of SBSAB when the samples are aged for both 85 min and 425 min. These results show that NA can delay the degradation effect of the SBS polymer inside SBS asphalt under different thermal-oxidative aging conditions.

It can be seen in Figure 9 that the second peaks of unaged NASBS are higher than that of unaged SBSAB, which means NA increases the molecular weight of asphaltene in unaged SBSAB. After undergoing the thermal-oxidative aging process, the second peaks of SBSAB and NASBS increase, which means that the molecular weight of asphaltene increases. This is because the polar groups in asphalt are combined into large molecular glue substances as the aging time increases. It is also obvious that the increasing extent of NASBS is lower than that of SBSAB, which reveals that NA could decrease the sensitivity of SBS asphalt to thermal-oxidative aging.

In order to make a better comparison of the contents of different components inside the asphalt samples under different aging levels, this section also adopts the area ratio of the main components to reveal the change pattern in the different components. The calculation of area ratios is conducted by integrating the corresponding area under the GPC test curves. The area ratios represent the content of the corresponding components inside asphalt.

**Figure 10:**
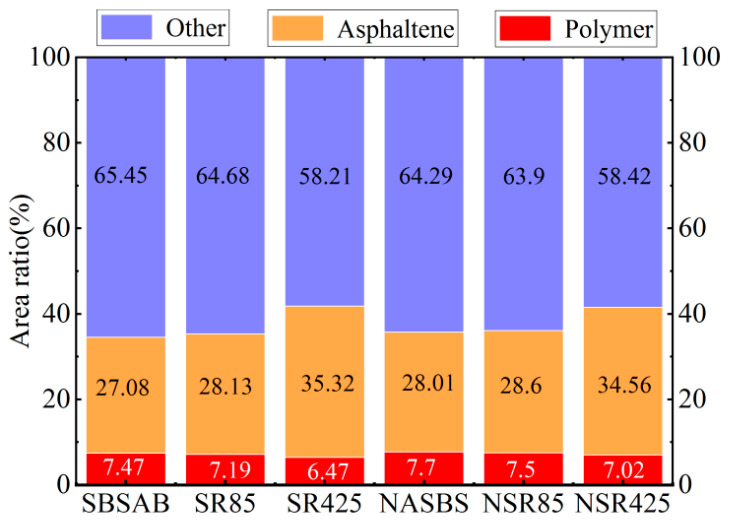
Area ratios of different components.

It can be seen in Figure 10 that the content of the SBS polymer in SBSAB decreases from 7.47% to 7.19% after 85 min of aging, and the decreasing extent is 3.75%. The corresponding decreasing degree of NASBS is 2.60%. The content of the SBS polymer in SBSAB decreases from 7.47% to 6.47% after 425 min of aging, and the decreasing extent is 13.39%. The corresponding decreasing degree of NASBS is 8.83% after undergoing 425 min of aging. 

The content of asphaltene in SBSAB increases from 27.08% to 28.13% after 85 min of aging, and the increasing extent is 3.88%. The corresponding increasing degree of NASBS is 2.11%. The content of asphaltene in SBSAB increases from 27.08% to 35.32% after 425 min of aging, and the increasing extent is 30.43%. The corresponding increasing degree of NASBS is 23.38% after undergoing 425 min of aging.

This paper also uses an index called weight-average molecular weight (*M_W_*) to represent the molecular weight of the SBS polymer and asphaltene inside different asphalt samples. *M_W_* is a statistically averaged molecular weight based on the molecular weights of a sample. The calculation method can be referenced from Equation (6).

(6) $$M_{w}=\sum_{i=1}^{n}\frac{w_{i}\times M_{i}}{w_{i}}$$

where *M_W_* is the average molecular weight and *w_i_* is the weight of molecular micelle *M_i_*.

**Figure 11:**
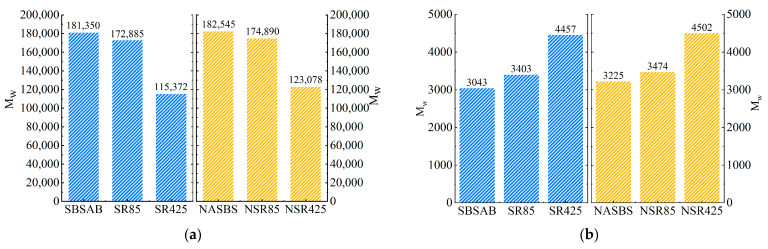
*M_W_* calculation result for (**a**) SBS polymer; (**b**) asphaltene.

As is shown in Figure 11, during the whole aging process, the *M_W_* of the SBS polymer inside different samples keeps decreasing. The decreasing extents of SBSAB after being aged for 85 min and 425 min are 4.67% and 36.38%, respectively. The related decreasing percentages of NASBS are 4.19% and 32.58%, respectively. 

The *M_W_* of asphaltene inside different samples keeps increasing as the aging time increases. The increasing extents of SBSAB after being aged for 85 min and 425 min are 11.83% and 46.47%, respectively. The related increasing percentages of NASBS are 7.72% and 39.60%, respectively. Therefore, it can be observed that the addition of NA delays the degradation of SBS polymers and the formation of macromolecular clusters under thermal-oxidative aging conditions [56]. Similarly, the conclusions drawn by Yan et al. support these findings [57].

#### 3.2.3. AFM Test Results

**Figure 12:**
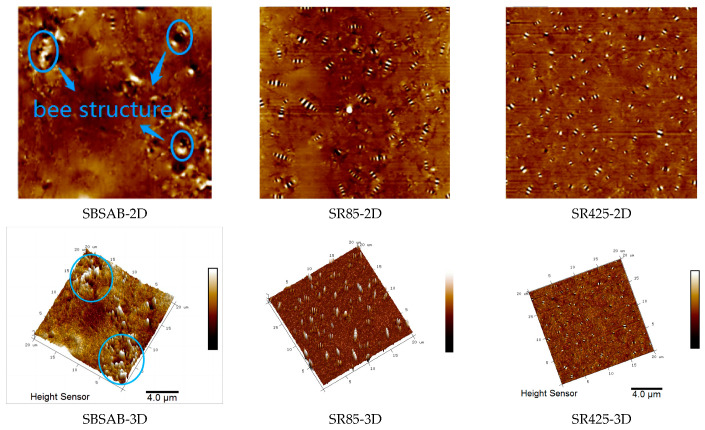
SBSAB micromorphology.

Figure 12 displays the 2D and 3D micromorphology of SBSAB under different aging times. The elliptical-shaped peaks marked in the figure are defined as the “bee structure”. Currently, relevant research suggests that asphaltene is the main component of “bee structures” [58,59]. It can be observed that after aging for 85 min, the quantity of “bee structures” in SBSAB significantly increases, and the volume of these structures also increases. After aging for 425 min, the quantity of “bee structures” further increases, but the volume starts to decrease. The increase in the quantity of “bee structures” is attributed to the conversion of small molecular components of asphalt into larger molecular components dominated by asphaltene clusters under the thermal-oxidative aging condition. After long-term aging, the quantity of asphaltene continues to increase, but the volume starts to decrease, indicating that asphaltene clusters begin to undergo fragmentation under long-term thermal-oxidative aging conditions. 

**Figure 13:**
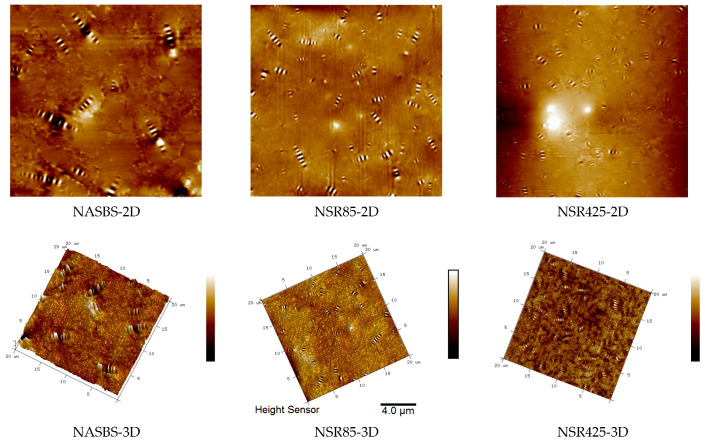
NASBS micromorphology.

Figure 13 displays the 2D and 3D micromorphology of NASBS under different aging times. After aging for 85 min, the quantity of “bee structures” significantly increases, but the volume decreases slightly. After aging for 425 min, there is no significant increase in the quantity of “bee structures”, but the volume decreases. By comparing the quantity and morphology changes in “bee structures” in the two asphalt binders, it can be concluded that NASBS with NA exhibits a more stable variation and stronger anti-aging ability under thermal-oxidative aging.

In order to make a clearer comparison of the atomic force microscope (AFM) test results for SBSAB and NASBS, this section adopts roughness to compare the change in micromorphology under different aging times. The calculation method for maximum roughness (*R_max_*) can be referenced from Equation (7).

(7) $$R_{max}=R_{P}+R_{V}$$

where *R_p_* is the height of the peak and *R_v_* is the height of the valley.

**Figure 14:**
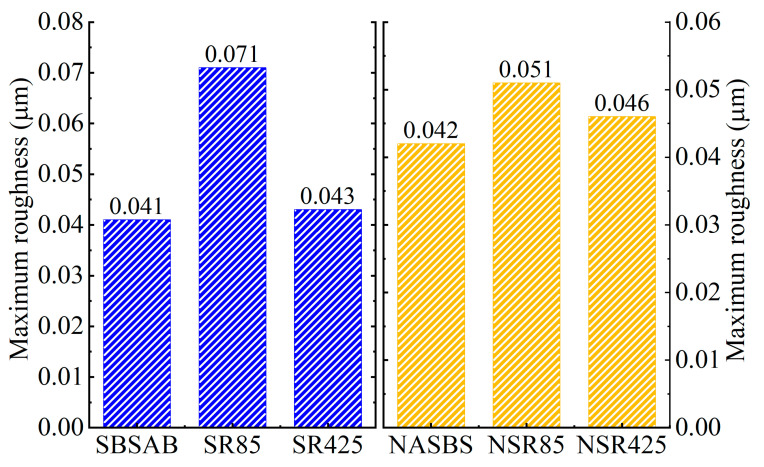
Roughness results.

It can be concluded from Figure 14 that after a short-term thermal-oxidative aging of 85 min, the roughness of SBSAB significantly increases. After undergoing 425 min of thermal-oxidative aging, the roughness decreases compared with the SBSAB aged for 85 min. This is because the content of asphaltene increases during the first 85 min of aging, but the asphaltene begins to fracture after 425 min of aging. The roughness of NASBS also increases under the influence of 85 min of thermal-oxidative aging, and it decreases when the aging time changes from 85 min to 425 min. The roughness maximum fluctuation extent of SBSAB is 73%, and that of NASBS is 21%. This indicates that under thermal-oxidative aging, the morphological changes in NASBS are more moderate compared to the morphological changes in SBSAB.

#### 3.2.4. Correlation Analysis between FTIR Indexes and Rheological Properties Indexes

FTIR test aging indexes have been used by other researchers to analyze the aging condition of asphalt materials and they are more accurate. Therefore, correlation analysis between the *I_B_*_/*S*_ and rheological property aging indexes is analyzed in this section. The data are listed in Table 3.

**Figure 15:**
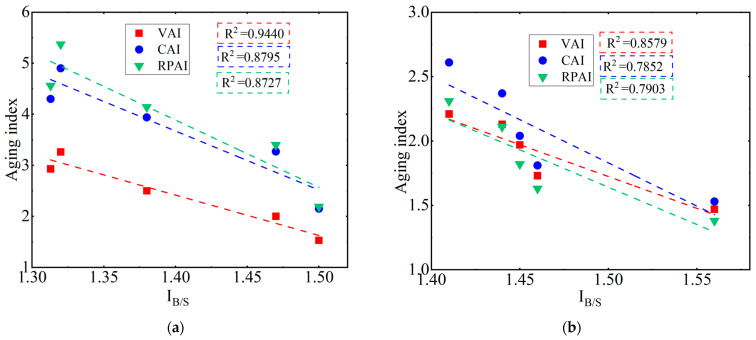
Correlation between the polymer index and rheological property aging indexes for (**a**) SBS asphalt; (**b**) NA/SBS modified asphalt.

The correlation analyzing the results between the *I_B_*_/*S*_ and rheological property aging indexes of SBSAB and NASBS is illustrated in Figure 15a and 15b, respectively. The correlation between the *I_B_*_/*S*_ and *VAI* is highest for SBSAB and NASBS, the decision coefficients (*R*^2^) between the *I_B_*_/*S*_ and *VAI* for SBSAB and NASBS being 0.9440 and 0.8579, respectively. The *VAI* represents the change in the flowability of the asphalt before and after thermal-oxidative aging. A lower *VAI* indicates better resistance to aging, and the *I_B_*_/*S*_ to some extent reflects the polymer content of aged asphalt. Therefore, better resistance to aging corresponds to a higher remaining polymer content after aging.

## 4. Conclusions

In this study, a rolling thin film oven test (RTFOT) was used to investigate the thermal-oxidative aging performance of SBSAB and NASBS asphalt binders under different aging times. The changes in the asphalt binder properties before and after thermal-oxidative aging were evaluated and compared through viscosity and DSR tests. Furthermore, the anti-aging mechanism of NA in SBS asphalt was analyzed from the perspectives of chemical functional groups, molecular weight, and microstructure, etc., using FTIR, GPC, and AFM tests, respectively. The following conclusions were drawn:

(1) Based on the rheological property aging indexes *VAI*, *CAI*, *RFAI* under different aging times, it was found that NASBS exhibited significantly improved anti-thermal-oxidative aging performance compared to SBSAB.

(2) For both short-term aging for 85 min and long-term aging for 425 min, continuous exposure to thermal-oxidative aging accelerated the aging of SBS asphalt. NA lessened the aging of SBS asphalt.

(3) An analysis of the FTIR results revealed that continuous thermal-oxidative aging increased sulfoxide groups and caused a continuous degradation of SBS polymers. The addition of NA delayed the growth of sulfoxide groups and the degradation of SBS polymers, thus improving the anti-aging performance of SBS asphalt.

(4) GPC tests showed that both SBSAB and NASBS experienced a significant increase in the content of asphaltene and a decrease in the content of SBS polymers during thermal-oxidative aging. The addition of NA slowed down the conversion of small molecules to large molecules in the asphalt and hindered the degradation of SBS polymers.

(5) The volume of the “bee structure” in NASBS decreases and the quantity increased with the duration of thermal-oxidative aging. On the other hand, the volume of the “bee structure” in SBSAB showed an initial increase followed by a decrease, with an overall increase in quantity. The change in roughness of NASBS was less affected by thermal-oxidative aging compared to that of SBSAB.

Based on a summary of the current literature, this paper studied the influence of NA on the thermal-oxidative aging characteristics of SBS-modified asphalt. Mechanism analyses were conducted to understand the excellent anti-aging mechanism of NA/SBS asphalt, filling the research gap in the current literature. The improvement of the anti-aging performance of asphalt corresponds to better fatigue performance [60]. The excellent anti-aging performance of NA/SBS asphalt can effectively alleviate the diseases caused by the aging of SBS asphalt pavement and contribute to the construction of aging-resistant asphalt pavement. In the future, further experimental and engineering research is needed to explore the fatigue life and economic benefits of NA/SBS asphalt.

## Figures and Tables

**Table 1:** Physical properties of the raw materials.

Material	Physical Property Index	Unit	Value or Characteristic
Base asphalt	Penetration (25 °C)	mm	6.7
	Softening point	°C	47
	Viscosity (135 °C)	Pa·s	0.52
	Ductility (10 °C)	mm	190
Nano-Al_2_O_3_	Grain size	nm	30
	Specific Surface Area	m^2^/g	40–60
	Appearance	/	White powder solid
SBS	Appearance	/	Linear leaf
	Average molecule weight	g/mol	110,000
	Styrene content	wt%	30

**Table 2:** Asphalt sample information and code.

Asphalt Type	Aging Level	Code
SBS modified asphalt	Unaged	SBSAB
	Aging for 85 min	SR85
	Aging for 170 min	SR170
	Aging for 255 min	SR255
	Aging for 340 min	SR340
	Aging for 425 min	SR425
Nano-Al_2_O_3_/SBS composite modified asphalt	Unaged	NASBS
	Aging for 85 min	NSR85
	Aging for 170 min	NSR170
	Aging for 255 min	NSR255
	Aging for 340 min	NSR340
	Aging for 425 min	NSR425

**Table 3:** *I_B_*_/*S*_ and rheological property aging indexes.

	Aging Time	*I_B_*_/*S*_	*VAI*	*CAI*	*RFAI*
SBSAB	85 min	1.5	1.53	2.15	2.19
	170 min	1.47	2.00	3.27	3.4
	255 min	1.38	2.5	3.94	4.14
	340 min	1.313	2.93	4.3	4.56
	425 min	1.32	3.26	4.9	5.37
NASBS	85 min	1.56	1.47	1.53	1.38
	170 min	1.46	1.73	1.81	1.63
	255 min	1.45	1.97	2.04	1.82
	340 min	1.44	2.13	2.37	2.11
	425 min	1.41	2.21	2.61	2.31

## Data Availability

The data presented in this study are available on request from the corresponding author.
